# Core-Shell Magnetic Gold Nanoparticles for Magnetic Field-Enhanced Radio-Photothermal Therapy in Cervical Cancer

**DOI:** 10.3390/nano7050111

**Published:** 2017-05-11

**Authors:** Rui Hu, Minxue Zheng, Jinchang Wu, Cheng Li, Danqing Shen, Dian Yang, Li Li, Mingfeng Ge, Zhimin Chang, Wenfei Dong

**Affiliations:** 1 CAS Key Laboratory of Bio-Medical Diagnostics, Suzhou Institute of Biomedical Engineering and Technology, Chinese Academy of Sciences, No. 88 Keling Road, Suzhou 215163, China; huuno@sohu.com (R.H.); minxue.zheng@sibet.ac.cn (M.Z.); 15251855931@163.com (D.Y.); jlu_li@163.com (L.L.); gemf@sibet.ac.cn (M.G.); 2 University of Chinese Academy of Sciences, Beijing 100049, China; 3 Department of Radiation Oncology, Affiliated Suzhou Hospital of Nanjing Medical University, Suzhou Municipal Hospital, No. 26 Daoqian RD, Suzhou 215000, China; wjinchang@sina.com (J.W.); chengli1981116@163.com (C.L.); tuzi.go@163.com (D.S.)

**Keywords:** cervical cancer, magnetic gold nanoparticles, synergetic, radio-photothermal therapy, magnetic field

## Abstract

The combination of radiotherapy (RT) and photothermal therapy (PTT) has been considered an attractive strategy in cervical cancer treatment. However, it remains a challenge to simultaneously enhance the radio-sensitivity of tumor tissue, develop tumor tissue-focused radiation therapies and combine dual therapeutic modalities. In this study, core-shell type magnetic gold (Fe_3_O_4_@Au) nanoparticles are exploited to achieve the synergistic efficacy of radio-photothermal therapy in cervical cancer. Fe_3_O_4_@Au nanoparticles (NPs) with uniform morphology exhibited superior surface plasmon resonance properties, excellent superparamagnetic properties, good biocompatibility and high photothermal conversion efficiency. For the in vitro tests, a low concentration of Fe_3_O_4_@Au NPs after a short period of near-infrared irradiation lead to the time-dependent death of cervical cancer cells. Further, the combination of RT and PTT induced synergistic anti-cancer effects in vitro. More importantly, an external magnetic field could significantly enhance the synergistic efficacy of Fe_3_O_4_@Au NPs by improving their internalization. Hence, the reported Fe_3_O_4_@Au NPs have the potential to be good nanoagents with excellent magnetic targeting ability for cervical cancer radio-photothermal treatment.

## 1. Introduction

Cervical cancer is the third most common cancer occurring in women all over the world, with approximately 500,000 new cases and approximately 300,000 deaths each year [1,2,3]. The clinical treatment procedures for cervical cancer include chemotherapy, radiation therapy, intracavitary or interstitial brachytherapy and even radical hysterectomy [4,5,6]. The patients’ different conditions, such as age, stage of the cancer, and tumor type, determine the different clinical therapeutic schemes [7,8,9]. Among them, radiotherapy is a prominent tool for many cervical cancer patients, including advanced stage cervical cancer patients and some earlier stage patients not suitable for surgery [10,11]. Although radiotherapy has been a major treatment in cervical cancer therapy, poor therapeutic effect and serious radiation-associated side effects such as cardiac toxicity, secondary malignancy, radiation pneumonitis, and lymphedema limit its clinical applications [12,13]. When ionizing radiation is applied to the tumor during radiotherapy, very little ionizing radiation energy is absorbed by the tumor tissue, while the normal tissues through which the irradiation beam passes are non-selectively damaged [14]. Therefore, it is urgent to enhance the sensitivity of tumor tissue to radiotherapy and achieve tumor cell-targeted radiotherapy in order to minimize the radiation dose delivered to surrounding healthy tissues, and to combine this approach with other safe treatment modalities to improve the therapeutic efficacy of treating cervical cancer. Currently, near-infrared (NIR) laser-induced photothermal therapy (PTT) has gained great interest due to its favorable biosafety [15]. PTT usually uses photo-absorbing agents to produce localized hyperpyrexia to ablate tumors with minimal invasiveness [16]. In addition, PTT is capable of potentiating radiotherapy via influencing the tumor microenvironment, increasing blood perfusion in tumors and reducing the hypoxic region and the subsequent radiation therapy-induced vascular disruption [17,18,19]. Hence, the synergistic treatment of radiotherapy and PTT is becoming an attractive approach for cervical cancer treatment. However, it is a challenge to seek an appropriate agent to synchronously improve the kill effect of two therapeutic modalities and enable targeting of the tumor to minimize the damage to normal tissue. 

With the fusion of biotechnology and nanotechnology, various nanomaterials have shown promising applications in cancer treatment [20,21,22]. Among them, gold nanoparticles have achieved great attention owing to their special properties, including versatile surface chemistry, good biocompatible and unique optical properties [23,24,25]. Gold nanoparticles are not only potential photothermal conversion agents for cancer photothermal therapy through surface plasma resonance processes, but they are also novel radiosensitizers due to their heightened photoelectric absorption efficacy and the improved electrons present [16,26]. Furthermore, gold nanoparticles of the appropriate size tend to localize at the tumor site via the enhanced permeability and retention (EPR) effect, a common nanoparticle delivery mechanism [27]. Passive tumor accumulation has been demonstrated to be facilitated by various target technologies [28,29]. In addition to passive targeting, magnetic targeting is among the most promising targeting technologies in the clinical treatment of tumors [30]. It is well known that a magnetic field can enhance the cellular uptake of nanoparticles containing magnetic materials [31,32]. Moreover, compared with other targeting technologies, a magnetic targeting strategy could be more suitable for clinical application because it is not only a more convenient way to improve agent accumulation at the tumor tissue, but it is also safer because the external stimulus strategy need not change the specific physical or chemical properties of the interior environment [33,34]. Hence, gold nanoparticles combined with magnetic material enable accumulation at a target region using a magnetic field for effective localized therapy at tumor sites, and would be advantageous agents for cervical cancer treatment. Among them, gold-coated magnetic nanoparticles are the most attractive nanoplatforms because the core-shell structure provides improved stabilization, good biocompatibility and surface reactivity. [35]. Many scientists have reported the fabrication of gold-coated magnetic nanoparticles and the application of these nanoparticles to detection, biomolecule immobilization, bio-separation and immunosensors [36,37,38,39]. However, there have been few studies on core-shell magnetic gold nanoparticles as radiosensitizers, and the potential of these nanoparticles in cervical cancer treatment has not been developed.

Herein, we designed core–shell magnetic gold (Fe_3_O_4_@Au) nanoparticles as both photothermal conversion materials and radiosensitizers. Fe_3_O_4_@Au nanoparticles (NPs) with uniform morphology showed a strong magnetic property and superior photothermal effect. Then, we studied the cytotoxicity and endocytosis of Fe_3_O_4_@Au NPs to cancer cells with or without a magnetic field. Finally, the efficacy of photothermal therapy, radiotherapy and synergetic photothermal-radiotherapy were evaluated in vitro. Our results established that Fe_3_O_4_@Au NPs are a promising platform for combined radio-photothermal therapy in cervical cancer.

## 2. Results and Discussion

### 2.1. Synthesis and Characterization

**Figure 1:**
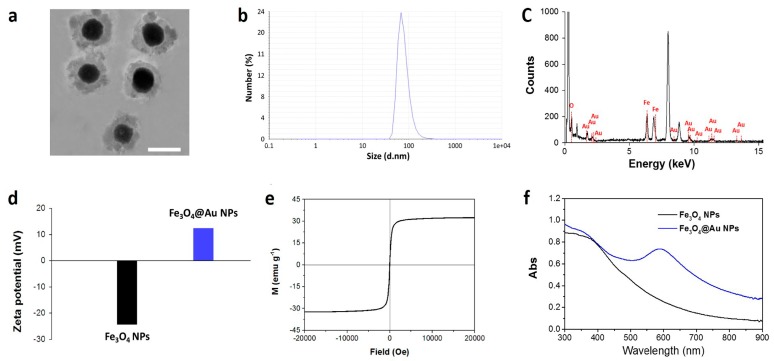
Characterization of Fe_3_O_4_@Au nanoparticles (NPs). (**a**) TEM images (scale bar: 100 nm); (**b**) Dynamic light scattering analysis of Fe_3_O_4_@Au NPs; (**c**) Energy dispersive X-ray spectrum of Fe_3_O_4_ NPs; (**d**) Zeta potential of Fe_3_O_4_ NPs and Fe_3_O_4_@Au NPs; (**e**) Magnetization curve of Fe_3_O_4_@Au NPs at 300 K; (**f**) UV-visible-near infrared (NIR) extinction spectrum of Fe_3_O_4_ NPs and Fe_3_O_4_@Au NPs.

Fe_3_O_4_@Au NPs were prepared by a single hydrothermal method. Briefly, Fe_3_O_4_ particles were dispersed in HAuCl_4_·4H_2_O solution by stirring to allow the adsorption of Au^3+^ onto the Fe_3_O_4_ surface. Then, NH_2_OH solution was added to reduce the Au^3+^, and core/shell Fe_3_O_4_@Au NPs were formed. As illustrated in Figure 1a, the TEM images of Fe_3_O_4_@Au NPs show uniform morphology and good dispersity. The Fe_3_O_4_ core was surrounded by a layer of gold shell with a thickness of ~20 nm, and the mean diameter of the produced Fe_3_O_4_@Au NPs was 100 nm by dynamic light scattering analysis (Figure 1b). The energy dispersive X-ray spectrum further demonstrated that the core/shell nanoparticles contained iron and oxygen, as shown in Figure 1c. Then, we detected the Zeta potential of Fe_3_O_4_ NPs and core-shell Fe_3_O_4_@Au NPs. As shown in Figure 1d, Fe_3_O_4_ NPs were negative and core-shell Fe3O4@Au NPs were positive after gold coating. Encouraged by the hybrid structure, we further explored the magnetic and optical properties of Fe_3_O_4_@Au NPs. As shown in Figure 1e, Fe_3_O_4_@Au NPs possess a superb magnetic response in aqueous solution with a saturation magnetization of approximately 30 emu/g, suggesting that the NPs enabled attraction in response to the extra magnetic field. The optical spectrum of the NPs is shown in Figure 1f. Pure Fe_3_O_4_ nanospheres exhibited very weak absorption in the visible-near infrared (NIR) spectrum. Notably, Fe_3_O_4_@Au NPs showed strong visible absorption at ~590 nm and significantly improved the NIR absorption at 808 nm. To explore the photoabsorption capability of Fe_3_O_4_@Au NPs at 808 nm, we calculated the extinction coefficient of Fe_3_O_4_@Au NPs according to the following equation [40]:

ε = A/LC = (AV_NC_ρN_A_)/(LC_wt_)

In this equation, A is the absorbance at 808 nm, L represents the path-length (1 cm), and C represents the molar concentration of Fe_3_O_4_@Au NPs. The molar concentration C can be calculated by C = C_wt_/V_NC_ρN_A_. C_wt_ is the weight concentration. N_A_ is Avogadro's constant. V_NC_ and ρ respectively represent the average volume and density of the Fe_3_O_4_-Au NPs. The calculated extinction coefficient ε was approximately 1.26 × 10^11^. The high extinction coefficient at 808 nm indicated that NIR light could induce a thermal effect in Fe_3_O_4_@Au NPs. 

### 2.2. Cell Uptake and Cytotoxicity of Fe_3_O_4_@Au NPs

**Figure 2:**
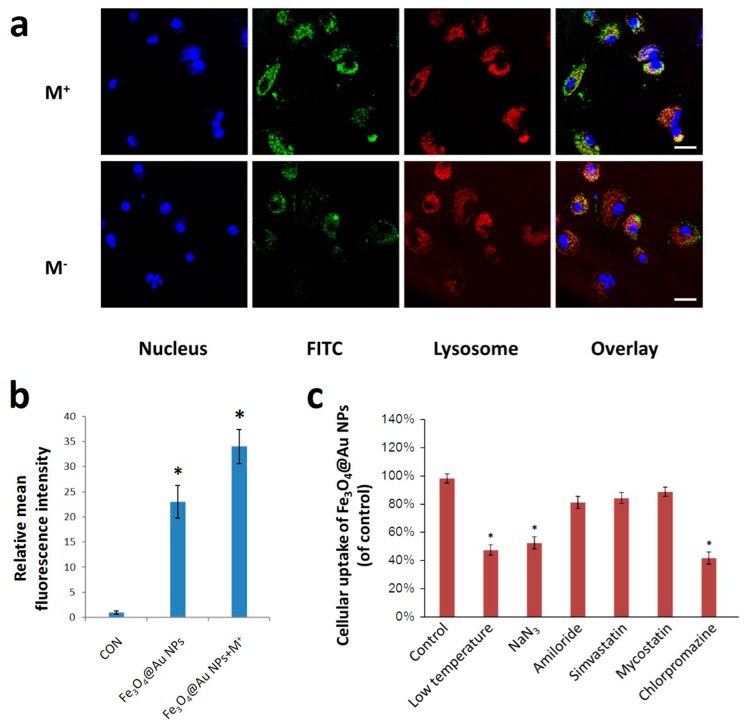
Cell uptake and cytotoxicity of Fe_3_O_4_@Au NPs. (**a**) Intracellular localization of endo/lysosomes (red) and Fe_3_O_4_@Au NPs (green) using confocal laser scanning microscopy images, (scale bar: 10 μm); (**b**) Quantitative analysis of fluorescence intensity of Fe_3_O_4_@Au NPs for 3 h with or without magnetic field; (**c**) Endocytic inhibition effect after treatment with various inhibitors. * *p* < 0.05 versus NP group.

To investigate the therapeutic potential of these NPs against cervical cancer, we first examined the endocytosis of Fe_3_O_4_@Au NPs into HeLa cells using confocal laser scanning microscope (CLSM)and flow cytometry. As shown in Figure 2a, green fluorescence represented fluorescein isothiocyanate (FITC)-labeled Fe_3_O_4_@Au NPs, and red fluorescence indicated lysosomes labeled by LysoTracker-Red. The partial overlay of green fluorescence and red fluorescence after 3 h incubation exhibited the co-localization of Fe_3_O_4_@Au NPs and lysosomes, which implied that these Fe_3_O_4_@Au NPs were endocytosed by lysosomes, and some Fe_3_O_4_@Au NPs entered the cytoplasm. More importantly, compared to the absence of a magnetic field, there was an obvious increase in the uptake of Fe_3_O_4_@Au NPs under the magnetic field. A similar endocytic behavior was observed by flow cytometry in Figure 2b. Quantitative analysis showed that the mean fluorescence intensity of Fe_3_O_4_@Au NPs under the magnetic field was clearly higher than without the magnetic field. These results demonstrated that Fe_3_O_4_@Au NPs exhibited superior endocytic properties and an external magnetic field could significantly enhance their cellular uptake behavior. To further explore the endocytosis mechanism of the Fe_3_O_4_@Au NPs under the magnetic field, various inhibitors were applied to block the cellular uptake pathways, and flow cytometry was utilized to quantitatively analyze the endocytic behavior. As shown in Figure 2c, the cellular uptake of Fe_3_O_4_@Au NPs dropped obviously in the presence of NaN_3_, low temperature and chlorpromazine. The results demonstrated that the clathrin-mediated pathway was the main cellular uptake pathway in the energy-dependent endocytosis of Fe_3_O_4_@Au NPs.

**Figure 3:**
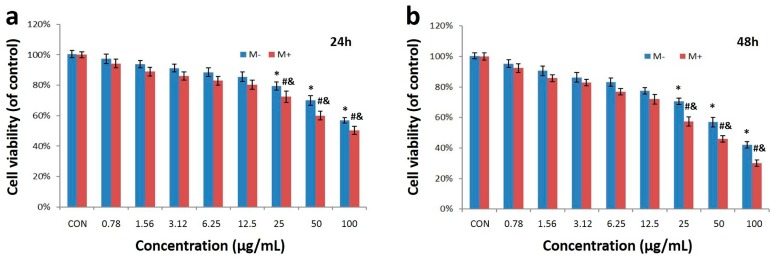
Cytotoxicity of Fe_3_O_4_@Au NPs to HeLa cells after 24 h (**a**) and 48 h (**b**). *, #, & *p* < 0.05 versus control group, & *p* < 0.05 versus non-magnetic treated-group.

To choose a safe dose of Fe_3_O_4_@Au NPs for further biomedical application, we assessed the cytotoxicity of Fe_3_O_4_@Au NPs at various concentrations (0.78, 1.56, 3.12, 6.25, 12.5, 25, 50 and 100 μg/mL) against human cervical cancer HeLa cells with or without the external magnetic field over 24 h and 48 h using the sulforhodamine B (SRB) assay. As shown in Figure 3a,b, dose-dependent cytotoxicity was observed for both groups. Furthermore, Fe_3_O_4_@Au NPs displayed a stronger cytotoxicity in the presence of external magnetic fields than outside magnetic fields: the magnetic field force exerted under the bottom side of the cell culture plates may enhance the endocytic ability of Fe_3_O_4_@Au NPs. The cell viability in both groups decreased significantly to 80% when the concentration was above the 12.5 μg/mL. Hence, considering the biosafety of the Fe_3_O_4_@Au NPs, 12.5 μg/mL was the optimum dose for further therapy against HeLa cells.

### 2.3. Photothermal Effect

**Figure 4:**
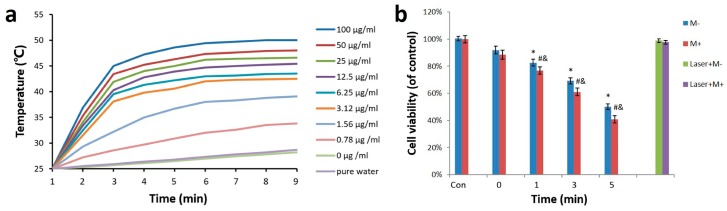
Photothermal conversion capacity of Fe_3_O_4_@Au NPs. (**a**) Temperature increase at different concentrations of Fe_3_O_4_@Au NPs after different irradiation times; (**b**) HeLa cell viability after incubation with Fe_3_O_4_@Au NPs for 0, 1, 3, and 5 min NIR irradiation. *, # *p* < 0.05 versus control group, & *p* < 0.05 versus non-magnetic group.

To evaluate the light heat conversion capacity of Fe_3_O_4_@Au NPs, the different concentrations (0–100 μg/mL) of NPs in the culture medium were examined under an 808-nm laser irradiation at a power density of 15 W/cm^2^. As illustrated in Figure 4a, the temperature of pure water and the blank cell culture medium showed only a 4 °C increase in 8 min, while the temperature of Fe_3_O_4_@Au NPs solution was obviously increased by 20 °C. The photothermal effects of the NPs were both time- and concentration-dependent. The temperature of 12.5 μg/mL NPs solution reached 43 °C under irradiation by NIR light after 3 min, which is critical for killing cancer cells. Fe_3_O_4_@Au NPs show excellent photothermal conversion capacity. To further verify the photothermal conversion capacity, we calculated the photothermal conversion efficiency *η* of Fe_3_O_4_@Au NPs at 808 nm, according to equation [41]:

$$\eta =\frac{Q_{\mathrm{NPs}}}{I\left(1-10^{-A}\right)}=\frac{Q-Q_{\mathrm{dis}}}{I\left(1-10^{-A}\right)}=\frac{hS\left(T_{\max}-T_{0}\right)-Q_{\mathrm{dis}}}{I\left(1-10^{-A}\right)}$$

I represents incident laser power, and A is the absorbance of Fe_3_O_4_@Au NPs at 808 nm. *Q*_NPs_ is heat dissipated by electron-phonon relaxation of the plasmons on the Fe_3_O_4_@Au NPs surface under 808 nm irradiation. *Q* is the heat conduction away from the system surface by air when the sample cell reaches the equilibrium temperature. *Q*_dis_ is the heat dissipation from light absorbed by the container itself, and the value of *Q*_dis_ was 5.4 mW according to the independent measure of a quartz cuvette cell with pure water. *S* is the surface area of the container, and h is the heat transfer coefficient. *T*_max_ is the ultimate temperature after radiation, and *T*_0_ is the initial temperature. The calculated photothermal conversion efficiency *η* of Fe_3_O_4_@Au NPs was 10.1%. Due to this high photothermal conversion capacity of Fe_3_O_4_@Au NPs, we believe that these NPs can be used as excellent PTT agents. 

Encouraged by the strong photothermal conversion capacity, we explored the photothermal effect of Fe_3_O_4_@Au NPs (12.5 μg/mL) on HeLa cell ablation for various irradiation times in the presence or absence of an external magnetic field by the SRB assay. As illustrated in Figure 4b, only less than 5% of HeLa cells were reduced in the NIR light alone group, even after 5 min of irradiation, compared to the control group. Nevertheless, after incubation with Fe_3_O_4_@Au NPs, the irradiation led to a time-dependent kill effect on HeLa cells. Almost 50% of the HeLa cells were dead after 5 min NIR irradiation without the magnetic field. Notably, with the aid of an external magnetic field, HeLa cells showed further cell death, reaching almost 60%. The results demonstrated that Fe_3_O_4_@Au NPs had a significant photothermal therapeutic effect on cervical cancer cells and the magnetic field was able to further enhance the photothermal ablation of tumor cells.

### 2.4. Photothermal-Radiotherapeutic Effect In Vitro

**Figure 5:**
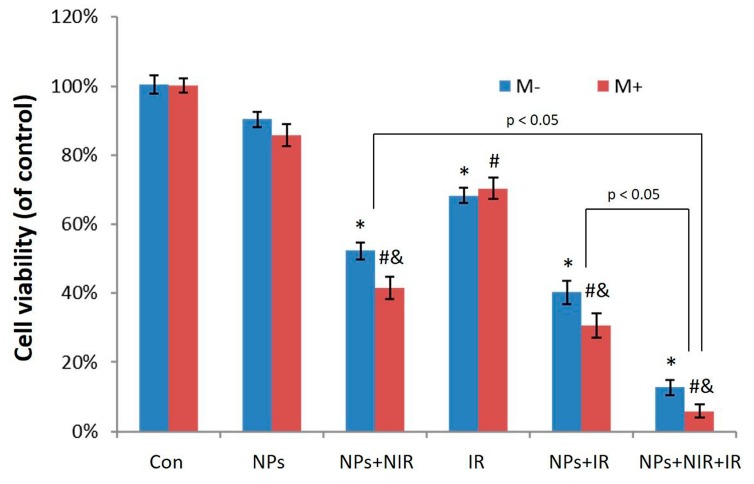
Cell viability of HeLa cells after different treatments. *, # *p* < 0.05 versus control group, & *p* < 0.05 versus non-magnetic group.

To determine the potential of Fe_3_O_4_@Au NPs as photothermal agents and radiosensitizers with a synergistic therapeutic effect, the combination of radiotherapy with photothermal therapy was then tested in vitro by the SRB assay. HeLa cells incubated with or without the NPs (12.5 μg/mL) for 12 h were irradiated by laser alone (15 W/cm^2^, 10 min), X-ray alone or laser combined with X-ray. As shown in Figure 5, Fe_3_O_4_@Au NPs did not cause substantial cell cytotoxicity without the assistance of NIR or X-ray. After X-ray radiation alone, the cell survival rate was 74.3%. When HeLa cells incubated with the NPs absorbed the same dose of X-ray radiation alone, the cell survival rate declined remarkably (40.2%). Interestingly, the same procedure in the presence of a magnetic field resulted in a comparatively high cytotoxicity (the cell survival rate was approximately 30.7%). More importantly, radiotherapy and photothermal therapy both inhibited HeLa cell growth, while combined photothermal-radio treatment was markedly superior to either RT alone or PTT alone. In the synergic group, most HeLa cells were damaged (the cell survival rate was 12.7%). It is worth noting that the combined treatment in the presence of a magnetic field was found to be more effective in destroying cancer cells than in the absence of a magnetic field. 

**Figure 6:**
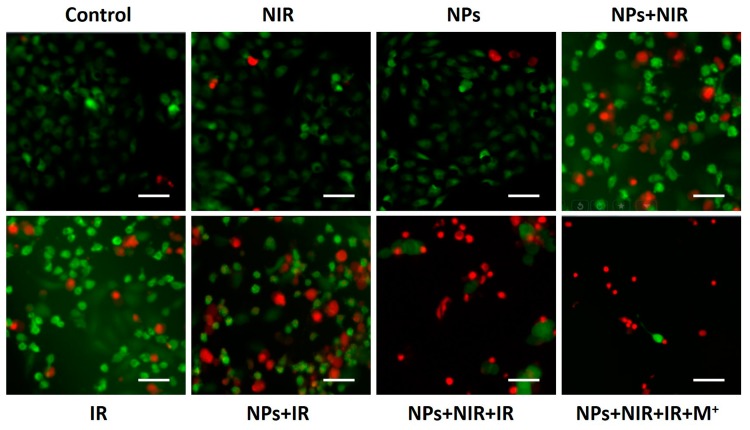
Live/Dead assay of HeLa cells after various treatment (scale bar: 50 mm).

Fluorescence images of calcein AM (green, live cells) and PI (red, dead cells) co-stained cells was used to further evaluate the efficacy of the combined photothermal-radio treatment. As shown in Figure 6, there was no obvious kill effect on HeLa cells in the NIR-treated group or the NP-treated group. Nevertheless, the treatment with NPs under the laser irradiation resulted in a part of cell death, confirming the effectiveness of PTT using Fe_3_O_4_@Au NPs. Compared with X-ray radiation alone, more red fluorescence was observed when HeLa cells were radiated after incubation with Fe_3_O_4_@Au NPs, which demonstrated that Fe_3_O_4_@Au NPs enhanced the sensibility of HeLa cells to RT. In combining the PTT/RT group, a large number of killed HeLa cells were detected. More importantly, the kill effect was further improved in presence of an extra magnetic field, which was consistent with the cell viability results. Collectively, these results confirmed that Fe_3_O_4_@Au NPs are a promising radiosensitizer with excellent photothermal properties. The combined PTT and RT triggered by Fe_3_O_4_@Au NPs enhanced the inhibitory effects of X-ray irradiation and showed a synergistically higher therapeutic efficacy for cervical cancer therapy, and the external magnetic stimulus significantly improved the combined therapeutic effect.

## 3. Materials and Methods 

### 3.1. Materials and Reagents

Iron (III) chloride anhydrous (FeCl_3_), tetrachloroauric acid (HAuCl_4_·3H_2_O), fluorescein isothiocyanate (FITC), DAPI and diethylene glycol (DEG) were bought from Sigma (St. Louis, MO, USA). RPMI-1640 medium and fetal bovine serum (FBS) were purchased from Beyotime Institute of Biotechnology (Haimen, Jiangsu, China). Sodium hydroxide and ethyl alcohol were obtained from Beijing Chemical Reagnet. All the reagents and materials could be bought and were not further purified before use.

### 3.2. Preparation of Fe_3_O_4_ Nanoparticles and Fe_3_O_4_@Au Nanoparticles

Fe_3_O_4_ NPs were synthesized via a high-temperature hydrolysis process. First, 2.0 g sodium hydroxide was added to 20 mL DEG at 120 °C for 1 h. Then, 0.8 mmol FeCl_3_ and 8 mmol PAA were added to 34 mL DEG and mixed in a three-necked flask with stirring. After 30 min, the mixture was heated to 265 °C under nitrogen protection. Then, 3.8 mL of DEG solution of NaOH was rapidly injected into the mixture and reacted for 1 h. Spherical Fe_3_O_4_ nanoparticles were obtained after washing by centrifugation.

Fe_3_O_4_@Au NPs were prepared using the reduction of gold iron with a hydroxylamine-coated Fe_3_O_4_ core. First, 1 mL of Fe_3_O_4_ solution (8.6 mg/mL) was injected into 0.1 mol/L HAuCl_4_·4H_2_O solution and stirred for 3 h. Then, 0.8 mL of ammonium hydroxide was added to the system. The mixture was heated to 40 °C for 1 h. The core-shell Fe_3_O_4_@Au nanoparticles were formed. In addition, FITC-labeled Fe_3_O_4_@Au NPs were synthesized using the same protocol, except that FITC (0.5 mg) was added to a solution of 0.1 mol/L HAuCl_4_·4H_2_O before being mixed with Fe_3_O_4_ nanoparticles in the dark.

### 3.3. Characterization of Core-Shell Fe_3_O_4_@Au NPs

The morphology and shape of core-shell Fe_3_O_4_@Au nanoparticles were characterized by TEM images (Suzhou University, Suzhou City, China)) with 200 kV. The size of Fe_3_O_4_@Au NPs was obtained on a ZetaPALS + BI-90Plus system. Magnetic properties were measured by VSM at 300 K. The photothermal effect of Fe_3_O_4_@Au NPs was examined by measuring the temperature of RPMI-1640 solution of Fe_3_O_4_@Au NPs at different concentrations (0, 0.78, 1.56, 3.12, 6.25, 12.5, 25, 50 and 100 μg/mL) under NIR laser irradiation (808 nm, 15 W/cm^2^) every 10 s.

### 3.4. Cell Uptake Test

HeLa cells were cultured on a clean 24-well plate at 2 × 10^4^ cells per well and maintained at 37 °C in RPMI-1640 medium with 10% (*v*/*v*) fetal bovine serum for 24 h. Then, FITC-labeled Fe_3_O_4_@Au NPs were co-cultured with HeLa cells for 3 h with or without cylindrical NdFeB permanent magnets (surface magnetic field of 0.2 T) placed under the 24-well plate. Then, DAPI (5 mg/mL) was used to stain the nuclei, and the cells were washed twice with PBS. A confocal laser scanning microscope (Olympus FV1000, C.L., Suzhou University, Suzhou, China) was used to observe the cell uptake of Fe_3_O_4_@Au NPs. Flow cytometry (C.L., Suzhou University, Suzhou, China) was utilized to quantitate the cell uptake of Fe_3_O_4_@Au NPs. Briefly, HeLa cells were cultured on a clean 24-well plate and co-cultured with FITC-labeled Fe_3_O_4_@Au for 24 h with or without a magnetic field. Then, the supernatant liquid was removed, and the cells were washed with PBS. The HeLa cells were resuspended through trypsinization, and the fluorescence content was examined by flow cytometer.

The endocytic mechanism was analyzed by treatment with various inhibitors to block the uptake pathway. For this purpose, 3.0 mg/mL NaN_3_ was used to deplete ATP, 20 µg/mL chlorpromazine was used to inhibit the clathrin-mediated pathway, 10 µg/mL simvastatin was used to inhibit the caveolae/lipid rafts pathway, 5 µg/mL mycostatin was used to inhibit the caveolae-mediated pathway, and 10 µg/mL of amiloride was used to inhibit the macropinocytosis pathway. The energy-dependent endocytosis was hindered by incubation at 4 °C. After incubation with the inhibitors for 30 min, the supernatant liquid with the inhibitors was removed, and the cells were washed with PBS and trypsinized. The flow cytometer was utilized to quantify the cell uptake of Fe_3_O_4_@Au.

### 3.5. In Vitro Cytotoxicity Assay

The inhibitory effects of each group on HeLa cells were evaluated through sulforhodamine B (SRB) assay. Briefly, 5 × 10^4^ cells were cultured on clean 96-well plates under standard culture conditions for 24 h. Then, the cells were treated with Fe_3_O_4_@Au NPs at various concentrations with or without NIR laser (808 nm, 15 W/cm^2^) and X-ray irradiation (5 Gy, 5 min) under or outside the magnetic field for 24 h and 48 h. Then, 0.4% (*w*/*v*) of sulforhodamine B was added to bind with the live cells protein. After washing by acetic acid and dissolution by Tris-base, the optical densityat 570 nm was examined, and the cell viability was calculated.

### 3.6. Statistical Analysis

All data were from three separate tests; experimental data were expressed as the mean ± SD. The data was analyzed by Student’s test and *p* < 0.05 indicated a difference in a statistically significant field.

## 4. Conclusions

In summary, we designed uniform core-shell type Fe_3_O_4_@Au NPs with a superb near-infrared absorption and excellent superparamagnetic property. The as-synthesized NPs exhibited low cytotoxicity, and the application of a magnetic field enhanced their endocytic ability. As potential photo-absorbing agents, Fe_3_O_4_@Au NPs show an excellent photothermal therapeutic effect against HeLa cells. When combined with X-ray radiation, the NPs could generate a synergistically radio-photothermal therapeutic effect. More importantly, these novel multifunctional NPs exhibited enhanced therapeutic efficacy with the aid of a magnetic field. This work highlights the potential of Fe_3_O_4_@Au NPs as a novel multifunctional nanoplatform for cervical cancer radio-photothermal treatment.
